# 

**Published:** 1884-10

**Authors:** 


### CONTENTS.

### COMMUNICATIONS.

Reflex Pain........................................................................ 473
Chemistry........................................................................ 479

### PROCEEDINGS.

Proceedings and Discussions of the Twenty-fourth Annual Meeting of the American Dental Association........................................ 486
Salmagundi........................................................................ 522

### EDITORIAL.

The Ohio State Dental Society........................................................ 525
Board of Examiners........................................................................ 526
Dentists' Benevolent Association........................................................ 527
Obituary........................................................................ 528

